# Gain modulation of probabilistic selection without synaptic relearning

**DOI:** 10.1371/journal.pone.0333350

**Published:** 2025-09-30

**Authors:** Elif Köksal-Ersöz, Pascal Chossat, Frédéric Lavigne

**Affiliations:** 1 Inria, Villeurbanne, France; 2 Cophy Team, Lyon Neuroscience Research Center, INSERM UMRS, CNRS UMR 5292, Université Claude Bernard Lyon 1, Bron, France; 3 MathNeuro Project-Team, Inria Branch of the University of Montpellier, Montpellier, France; 4 Université Côte d’Azur, Laboratoire Jean-Alexandre Dieudonné, Nice, France; 5 Université Côte d’Azur, CNRS, BCL, France; Indiana University Purdue University Indianapolis, UNITED STATES OF AMERICA

## Abstract

Adaptation of behavior requires the brain to change goals in a changing environment. Synaptic learning has shown its effectiveness in changing the probability of selecting actions based on their outcome. In the extreme case, it is vital not to repeat an action to a given goal that led to harmful punishment. The present model proposes a simple neural mechanism of gain modulation that makes possible immediate changes in the probability of selecting a goal after punishment of variable intensity. The results show how gain modulation determines the type of elementary navigation process within the state space of a network of neuronal populations of excitatory neurons regulated by inhibition. Immediately after punishment, the system can avoid the punished populations by going back or jumping to unpunished populations. This does not require particular credit assignment at the ‘choice’ population (the branching node) but only modulation of the gain of units active at the time of punishment (at the end of the punished branch). In this way, gain modulation encodes memories of past experiences that change behavior without modification of synaptic efficacies. This neuronal non-synaptic learning mechanism does not require statistical relearning. It helps the system not repeat harmful choices that may lead to further punishments. Thus, such a neuronal learning mechanism can complement synaptic plasticity.

## Introduction

Adaptation of behavior requires the brain to select actions that bring benefits and avoid those that bring costs. The selection of the most likely rewarded action requires updating the relationship between actions and their rewards and punishments from past experience [1–3]. In a stable environment, the probability of the outcomes is best estimated by experiences going back a long way to ensure exploitation of the rewarded actions. If the probabilistic structure of the feedback changes, actions become prone to errors due to uncertainty on the expected feedback. The exploration of different actions and the relearning must then update the probability of action-outcome [4, 5] by statistical learning of trials and errors [6–8]. This raises the question of the degree of recency of the experiences and of the intensity of the outcomes to be taken into account.

Animal studies report that both ancient and recent rewards are memorized [9–11]. Changes in the rate of rewards and punishments can be adjusted by changes in a single learning rate to update synaptic efficacies [3, 10, 12, 13]. Computational models have investigated a learning rate in synapses connecting a context to different actions that depends on the magnitude of the error signal to optimize the weighting of old and recent experiences over multiple timescales [14, 15]. When changes in the environment are transient, different structures of action-feedback are associated to transiently alternating contexts. In this case, it is beneficial to adapt behaviors to the transient state without forgetting the previous state, and hence without the need to relearn it. Synaptic relearning leads to forgetting the previous probabilistic structure of action-outcome relations. The previously learned and forgotten environment has to be learned again through repetition of trials and errors, even at a fast rate. However, even more dramatically, in the extreme case of severe and dangerous punishment, the action that led to it should not be repeated. Some errors should not be made twice. This does not give time (or opportunity) for statistical relearning. Then is it possible to change actions without synaptic relearning? And in the extreme case, without the need for any further learning trial?

Various cortical functions have been reported to depend on modulation of input-output gain at the level of neurons, defined as the slope of the transfer function of neurons [16–20]. At the level of network behavior, gain modulation changes the dynamics of large-scale networks [21] and the correlation of neuronal output activity [22], giving the network the computational ability to change which neurons are activated by a context even though values of synaptic efficacy are fixed [23]. In addition, a recent computational model has reported that a network can switch activation between sequences of neurons that coding for items depending on the modulation of the gain of these neurons [24]. Gain modulation was shown to be efficient in changing the probability of activating an item or another in the network state space for a fixed synaptic matrix [24]. However, the pure effects of gain, *i.e.,* for fixed synaptic values, on choice behavior is still an open question. Here we investigate the conditions under which neuronal gain alone enables switching from one choice to another without the need for synaptic relearning, and possibly with immediate effect in case of punishment. This raises the question of the way to assign punishment to the sequence of neurons that led to the punished behavior (*e.g.,* [25–29]).

## Methods

The model has been directly inspired by [24], where the retrieval of multiple sequences in a collection of *P* learned states $\xi^{1},\cdots ,\xi^{P}$ has been investigated using the framework introduced in [30, 31]. In this model, the network contains *N*>*P* ‘units’ (mesoscale populations of highly interconnected neurons with nearly identical properties). Each learned state in the network is a dynamically stable ‘pattern’ made of two active units, the other units being inactive. Moreover, these patterns can be destabilized under the effect of short-term synaptic depression (STD), allowing for the dynamics of activation of patterns in the network state space.

In [30, 31], two consecutive learned patterns share one unit, so that the patterns form a chain of overlapping states $\xi^{1}\;-\;\xi^{2}\;-\;\cdots \;-\;\xi^{P}$. For example, pattern $\xi^{1}$ has active units 1 and 2 while pattern $\xi^{2}$ has active units 2 and 3, etc. The minimum number of units to form a chain is *N* = *P* + 1. The synaptic matrix was constructed within a minimal setting: two units encoding for one pattern, and one common unit between the pattern. Increasing the number of units per pattern and/or the number of overlapping units would not qualitatively change the network behavior as long as the patterns are attractor states of the network and the parameters ensure their sequential activation. It has been shown that, under the combined effect of STD and noise, the overlapping condition allows one to produce a sequential stochastic dynamics, one state $\xi^{i}$ “jumping" to $\xi^{i+1}$ with high probability (so-called *latching dynamics*). The basic idea is that when a unit is active, its synapses toward post-synaptic units as well as the autosynapse toward itself slowly depress due to STD. The level of STD depends on the activity of the unit, higher for the activated unit than for the post-synaptic unit that is not yet fully activated, and on the level of potentiation of the synapse, higher for the autosynapse than for the synapses toward post-synaptic units. Taken together, these effects of STD reduce autoactivation of the pre-synaptic unit more than the activation of post-synaptic units. As a consequence, post-synaptic units become more activated than the pre-synaptic unit, leading to the activation, step by step, of a sequence of units.

In [24] we further analyzed the case where, as the system starting from $\xi^{1}$ reaches a given state $\xi^{m}$ in the chain, it faces a choice among several continuing branches. In the simplest case, as considered in the present work, the chain splits at $\xi^{m}$ into two chains, which we index as follows: $\xi^{1}\;-\;\xi^{2}\cdots \;-\;\xi^{m}$ is the initial branch (branch 0), $\xi^{m+1}\;-\;\cdots \;-\;\xi^{q}$ is branch 1, and $\xi^{q+1}\;-\;\cdots \;-\;\xi^{P}$ is branch 2. This implies that the unit m+1 is shared by the three patterns $\xi^{m}$, $\xi^{m\;+\;1}$ and $\xi^{q+1}$ (branching node).

As shown in [24], as long as the connectivity matrix is symmetric and the weights of connections from $\xi^{m}$ to $\xi^{m+1}$ and to $\xi^{q+1}$ are equal, the probabilities that the dynamics starting from $\xi^{1}$ continues in branch 1 or branch 2 are equal, but whenever the weights of connections from $\xi^{m}$ to $\xi^{m+1}$ are greater than those from $\xi^{m}$ to $\xi^{q+1}$, the probability of continuing in branch 1 is larger than the probability of continuing in branch 2.

This is the context of the present work: due to a reward (for example) delivered each time the system has engaged on branch 1 during the learning phase, the probability to choose branch 1 is higher that that of choosing branch 2. Assume now that as the system reaches a certain pattern in branch 1, it receives a punishment that produces a sudden drop of the gain of the corresponding active units. The question is where does it go next? Does it stay on the same branch or switch to branch 0 or 2? This is the basic question which is asked in this paper.

Let us now introduce our model and give a brief description of how it does produce latching dynamics, referring to [24] for details. The equations for the units are derived from the conventional equations for neural masses (homogeneous populations of nearly identical neurons) in which we have replaced the membrane potential *u*_*i*_ of each unit *i* by the activity $x_{i}=S(u_{i})=1/(1\;+\;e^{-\gamma u_{i}})$. The advantage of this formulation is that it reveals a geometric structure in the equations which in certain cases, and certainly in the present work, simplifies and lightens the analysis of the system. Indeed, the variables are now the activities, which take values in the interval [0,1] after the term $S^{-1}(x_{i})$, which appears in the equations for *x*_*i*_’s, has been replaced by its polynomial expansion (we chose the simplest linear approximation). Then if *x*_*i*_ is set to 0 (inactive state) or 1 (active state) at initial time, it remains fixed at that value for all time, as can be seen on Eq (1) for unit *i*.

$$\dot{x_{i}}=x_{i}(1-x_{i})\left(-\frac{4}{\gamma }x_{i}+\sum_{j=1}^{N}J_{i,j}x_{j}-\lambda \sum_{j=1}^{N}x_{j}-\lambda \nu_{i}x_{i}-I+\frac{2}{\gamma }\right)+\eta$$ (1)

where *γ* is the gain and *λ* is the inhibitory coefficient. The effects of inhibition within the network are modeled by a term proportional to the average activity [32–34]. The coefficient $\nu_{i}$ accounts for the possibility of short-range inhibitory loops between excitatory neurons and inhibitory interneurons. This allows for selective self-inhibition of excitatory neurons, which regulates excitation proportionally to the number of excitatory afferent connections to the excited units. In our case, we take $\nu_{i}=0$ for i≠m+1 (note that *m* is the index of the branching node in the network) and $\nu_{m\;+\;1}=1$ (unit m+1 receives inputs from units m,m+1 and q+1). The term *I* stands for a constant, global inhibition. Finally, *η* is a white Gaussian noise term. Note that *x*_*i*_ = 0 or 1 are always solutions of (1). Hence, any state such that *x*_*i*_ = 0 or 1 for all i=1…,N is an equilibrium (or steady-state) of the system. The learned states in our model are stable solutions of this type.

The STD is expressed as follows. Let *J*_*ij*_(*t*) be the strength of the connection from units *j* to *i* at time *t*. We write $J_{ij}(t)=J_{ij}^{max}s_{j}(t)$, where $J_{ij}^{max}$ are the connectivity coefficients (synaptic efficacies) resulting from the learning process and *s*_*j*_(*t*) follow the STD law given in [35], which is equivalent to

$$\tau_{r}\dot{s_{i}}=1-s_{i}-\rho s_{i}x_{i}.$$ (2)

where $\tau_{r}$ is the time constant of the STD and *ρ* relates to the fraction of available resources in the synapse. The weights $J_{ij}^{max}$ are calculated from the simple symmetric Hebbian rule $J_{ij}^{max}=\sum_{k}\xi_{i}^{k}\xi_{j}^{k}$ but other symmetric rules could also be used.

**Fig 1:**
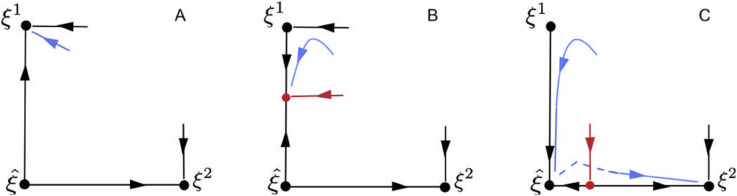
Three snapshots of the “latching” dynamics. Before destabilization of the first learned state $\xi^{1}$ (A), intermediate configuration with bifurcated equilibrium (B) and after coalescence of the bifurcated equilibrium with the state $\hat{\xi }$ (C). The trajectory starting close to $\xi^{1}$ (blue arrow) follows the bifurcated equilibrium point (red dot) after $\xi^{1}$ becomes unstable (from A to B). The dotted line sketches the stochastic jump over the basin of attraction of $\hat{\xi }$ towards the basin of attraction of $\xi^{2}$ (C). The red arrow shows the transverse stability of the red equilibrium point. It also indicates the boundary between the bassins of attraction of $\hat{\xi }$ and $\xi^{2}$ in the right panel.

In this framework the production of sequences by latching dynamics is a robust phenomenon, which can be illustrated by considering the case of two consecutive stable states (patterns) $\xi^{1}=(1,1,0)$ and $\xi^{2}=(0,1,1)$ in a system of three units with activities $\left(x_{1},x_{2},x_{3}\right)$. Therefore, $\xi^{1}$ and $\xi^{2}$ share the second excited unit, and we set $\hat{\xi }=(0,1,0)$ for the overlapping state. This generalizes to an arbitrary number of states (see [31]). Initially, the system lies near $\xi^{1}$. However, after some time, STD destabilizes it along *x*_1_. Due to the invariance properties of (1), the relevant dynamics lies in the face *x*_2_ = 1 of the cube $0\leq x_{1},x_{2},x_{3}\leq 1$, to which we now restrict our attention. The STD dynamics is slow compared to the dynamics of neurons activity: it can be shown that in the slow limit it acts as if synaptic efficacies, in particular *s*_1_, were a free parameter which initially equals 1 and which we move downward “by hand”. Then Fig 1 sketches the phenomenon. It shows three consecutive snapshots of the evolution of the system. The sketch on the left corresponds to the case where $\xi^{1}$ has not yet been destabilized by STD. The middle drawing shows what happens after destabilization: a bifurcated stable state (red point) appears on the edge connecting $\xi^{1}$ to $\hat{\xi }$. The blue line shows the nearby dynamics. This is a *dynamic bifurcation*. The dynamics follows this bifurcated state along the edge, until it reaches $\hat{\xi }$. This produces a new bifurcation that leads to the appearance of an unstable equilibrium on the edge connecting $\hat{\xi }$ to $\xi^{2}$ (sketch on the right) while $\hat{\xi }$ has become stable, so that in the absence of noise the dynamics would converge to $\hat{\xi }$. However, as long as this unstable equilibrium stays close to $\hat{\xi }$, a limited amount of noise suffices to allow the system to “jump” above it and to converge towards $\xi^{2}$ (this is illustrated by the dotted blue line).

*Remark 1.* A slightly different scenario may occur when the gain *γ* is strong enough, in which case stronger noise is required and *regular sequences* (meaning, sequences which follow the order $\xi^{i}\to \xi^{i+1}$) are likely to be shorter; see [31] for details.

*Remark 2.* With the Hebb rule that we consider here, the latching dynamics stops at pattern $\xi^{P-1}=\xi^{N-2}$ because the autosynaptic strength $J_{NN}^{max}$ of the last unit is not sufficient to have it excited. This is an artifact due to the choice *P* = *N*−1 for the number of learned patterns and will not affect subsequent simulations and analysis.

Now suppose that in the course of latching dynamics, a sudden decrease occurs for the gain of a unit while this unit is being excited. This may happen because some punishment is applied when the corresponding learned pattern is reached. This is a discontinuous process that can radically modify the dynamics in the following way. Let us look at Fig 1 and assume that the punished pattern is $\xi^{2}$. The drop in gain value at $\xi^{2}$ shifts the red equilibrium to the right on the axis $\hat{\xi }\;-\;\xi^{2}$, so that the stochastic jump from $\hat{\xi }$ to $\xi^{2}$ will require longer time or stronger noise. If the drop is large enough $\xi^{2}$ will become unstable and unreachable at all.

In the next section we numerically investigate this phenomenon in the case when an initial chain (branch 0) splits into two branches, one of them (branch 1) being more likely under latching dynamics because reward was given during learning process. As the last reachable pattern (see Remark 2) on branch 1 becomes excited, a punishment is applied as explained above, and we observe what the system does next.

For these simulations, we took parameter values of Eqs (1)–(2) from [24, 31], namely λ=0.6, I=2/γ, $\nu_{4}=1$, η=𝒩(0,0.04), ρ=1.2, $\tau_{r}=300$, γ=10 (without punishment), unless otherwise stated. We consider 4 different strengths of punishment, with corresponding gain taking values resp. γ={9,5,3.3,2.5}, that is from weak (10%) to strong (75%) punishment rates. For each parameter combination, 1000 simulations were realized using the Euler-Maruyama method with time steps of 0.01 ms.

## Results and discussion

**Fig 2:**
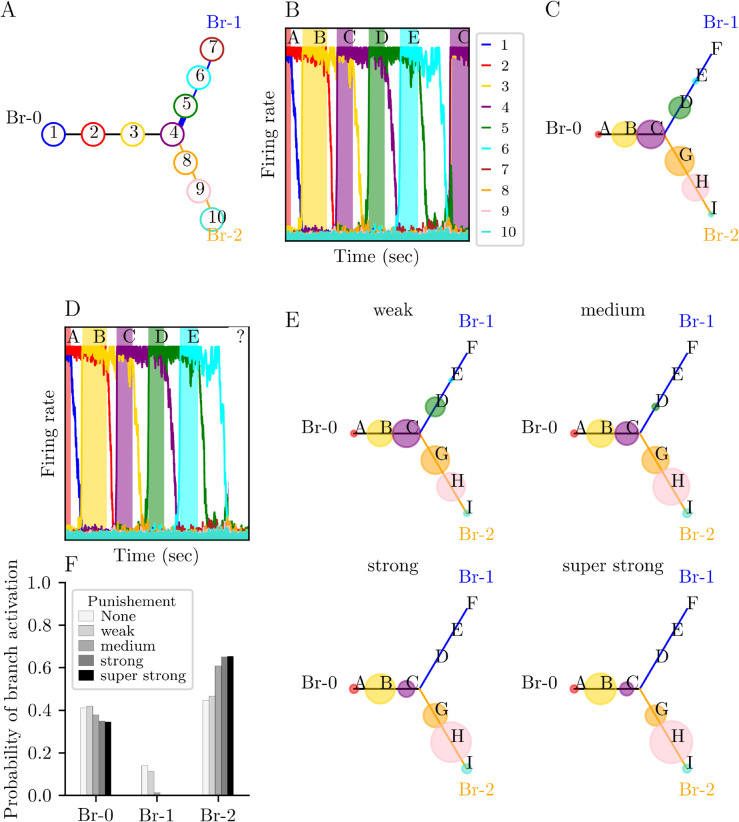
Branching behavior of a N = 10 units network at Trial T (case of strong inhibition λ = 0.60) (A) Network architecture of 10 neuronal units (represented by numbered circles). Unit 4 is the branching node between three branches 0, 1 and 2. The synaptic efficacy between units 4 and 5 (from branch 0 to branch 1) is 10% stronger than between units 4 and 8 (from branch 0 to branch 2). (B) Network behavior is described from the activation of the starting units 1 and 2, at the beginning of the branch 0 (Br-0). Pairs of directly adjacent units correspond to patterns that have one unit overlap, described by letters from A to I. Note that the branching unit 4 is part of patterns C, D, and G, themselves parts of branches 0, 1 and 2, respectively (see Method). In this example, activation propagates from units 1 to 4 in a sequence and continues to units 5 and 6 in branch 1 (Br-1). After a regular sequence of units that ends with pattern E in Br-1, the network breaks the regular sequence and jumps directly to another pattern, here C (units 3 and 4) in the example. (C) The same network as in panel (A) where the 9 patterns are displayed instead of units (colored circles). Circles size is proportional to the probability of activation of the patterns following pattern E, that is after a regular sequence from the starting pattern A to pattern E, as demonstrated in panel (B). (D) We ask how the network will react to the punishment that is given on pattern E and what will be the first activated pattern (E) Probability of activation of the patterns (circles size) in the 3 branches immediately after the punished pattern E during the punished trial *T*, as a function of the level of punishment. (F) Pattern activation given in panels (C) and (D) is regrouped into corresponding three branches. When the punishment is weak (10% decrease in neuronal gain), the system can still activate patterns D or E but in only 11% of the trials. For a medium punishment (50% decrease in gain), this ratio decreases to 1%. Whereas for a strong punishment (66% decrease in gain), the system does not activate patterns along Br-1 anymore. Instead, the system switch branch by activating patterns along branch 2 (Br-2) with a 40% increase from weak to strong punishment.

The model presented here allows mathematical analysis and simulation of the effect of gain modulation on the selection of goals towards which to direct actions. We investigated a network of *N* = 10 units encoding 9 learned patterns $\xi^{1},\ldots ,\xi^{9}$. Notice that this is a minimal configuration to observe the impact of punishment on the branch choice. The three units (*m* = 3) before the branching unit (m+1=4) prevent the impact of the transient dynamic branch encoding for two patterns before the branching unit *x*_4_. If the punishment pattern was encoded by the branching unit, the punishment signal would affect activity along the initial branch. Since we wondered about the impact on choices, we included at least one unit between the punished and branching units. Since the units at the endpoints of the chains encode only one pattern, they are less excited than the middle units, which encode two patterns. However, those units are essential for the activation of the pattern just before (see Remark 2 above). Therefore, we had three units after the branching unit with q=m+1+3=7. Under these constraints, we obtained a minimal network of *N* = 10 units (three units per branch plus the branching unit) that encodes P=N−1=9 patterns, which share one unit in common. The units are placed in a 3-node graph where units 1-3 are along branch 0, units 5-7 along branch 1, units 8-10 along branch 2 and unit 4 is the branching node connected to 3, 5 and 8 (Fig 2A). For convenience we will use from now on the alphabetical notation A,B, , I for these patterns (see e.g. Fig 2B).

The activation of two units side by side (*e.g.*, *x*_2_ and *x*_3_) generates a pattern corresponding to a network state (*e.g.*, B). The branching synaptic architecture corresponds to a classical Y-maze in which, from a starting branch, a choice between two branches leads to rewards or punishments [36]. Here, the network embeds patterns (pairs of neurons populations) that code for successive “places to go” to reach the final goals at the end of the branches. A change in the activated pattern corresponds to the activation of a new “place to go” where to orient actions in the Y-maze. For the sake of clarity, we focus here on the elementary building block of network behavior at a single branching node, but the results presented here can be generalized to more complex networks involving 4-way or more branchings as well as *branches in branches*).

The synaptic coupling coefficient between units 4 and 5 (branch 1) was 10% stronger than between units 3 and 4 (branch 0), and units 4 and 8 (branch 2) [24]. We study whether gain modulation can change the sequence of units activated during a punished trial *T* and during the following trial T+1. To clearly identify pure effects of gain on the network behavior, gain was changed by keeping synaptic efficacies constant, that is stronger efficacy between units 4 and 5 (toward branch 1) than between units 4 and 8 (toward branch 2; Fig 2A). For simulations, the system was initialized in pattern A and punishment was applied to units 5 and 6 coding for pattern E at the end of the branch when it became activated during the trial *T*. In this way, punishment was assigned only to neurons active at the time of feedback and not to the entire sequence of units that were activated before punishment [26]. Changes in gain are assumed to depend on punishment signaling (noradrenalin, serotonin and/or dopamine; [37–41] that is reported to decrease neuronal gain [42–44, 49]. The results show that punishment-dependent gain modulation changes the probability of activating the punished *vs.* unpunished patterns. In particular, the activations of patterns D and E decrease (Fig 2E, 2F).

**Fig 3:**
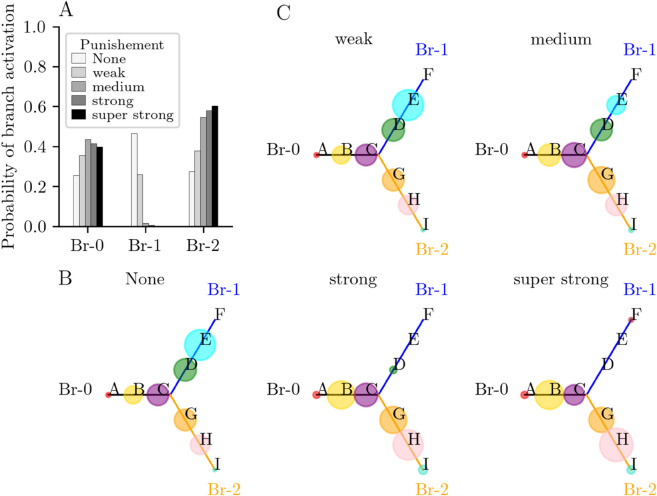
Branching behavior of a N = 10 units network at trial T (case of weak inhibition λ = 0.55). (A) Probability of activation of the 3 branches (any pattern) immediately after the punished pattern E activated during a regular sequence A-B-C-D-E at trial T. Network behavior depends on the level of punishment (circle size is proportional to the probability of activation of the patterns following pattern E). In the absence of any punishment (‘None’), the system takes branch 1 (Br-1) in 46% of the trials. When the punishment is weak, the system activates Br-1 in 0.26% the trials. This ratio decreases to 1% for a medium punishment and to 0 strong punishment. Instead, the system activates patterns along branch 2 (Br-2) with a 25% increase from weak to strong punishment. Pattern activity is detailed in (B) and (C). (B) The probability of activation of the patterns after deactivation of pattern E in the absence of punishment. (C) The probability of activation of the patterns after deactivation of pattern E as a function of punishment rate.

We investigated further the system’s robustness to variations in the short-term depression recovery time constant ($\tau_{r}$) and the global activity-dependent inhibition (*λ*). Decreasing activity-dependent global inhibition induces reactivation of patterns E and D in branch 1 for weak and medium levels of punishment, suggesting that the system persists in the punished action (see Fig 3 for λ=0.55). Branch preference does not depend on the global inhibition for strong punishment rates for which the system avoids branch 1 and goes to branch 0 or branch 2. Changing the recovery time constant $\tau_{r}$ does not change the global behavior of the system in response to punishment, that is, increasing the punishment rate decreases the activation probability of branch 1, and the activation probability of branch 2 increases for slow synapses in particular (S1 Fig). We also observe a slight increase in branch 2 preference with global inhibition and recovery time constant.

**Fig 4:**
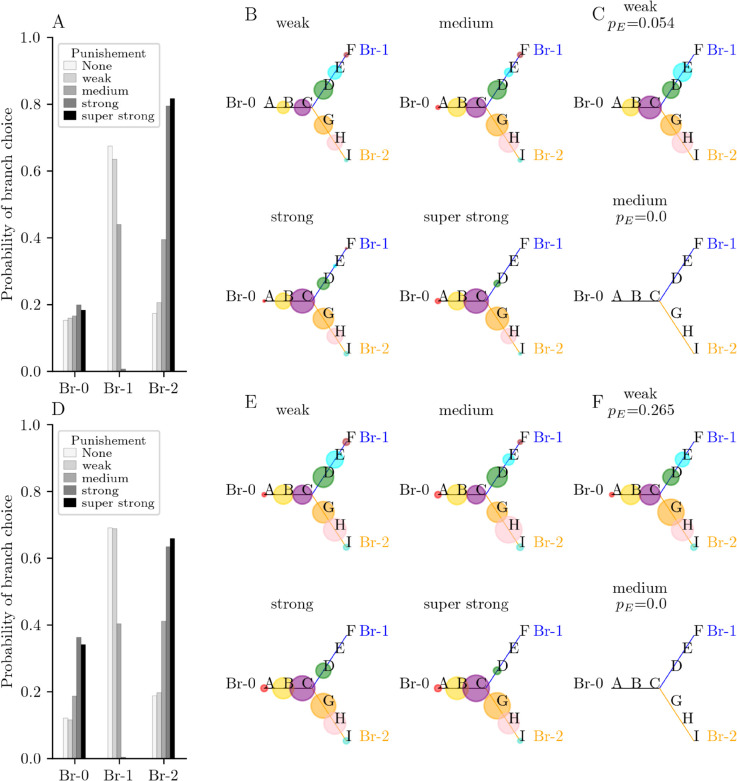
Branching behavior of a N = 10 units network at Trial T + 1. (A-C) Case of weak inhibition (λ=0.55). (A) Probability of choosing the branches from starting pattern A during trial T+1. In the absence of any punishment (‘None’), the system takes branch 1 (Br-1) in 64% of the trials. For a weak punishment, the system activates Br-1 in 55% of trials and branch 2 (Br-2) in 20% of trials. Moderate punishment equalizes the probability between the two branches (42% for Br-1 and 39% for Br-2). For strong punishment, the networks activates the Br-2 only (0% for Br-1 vs 80% for Br-2). (B) Probability of activation of the patterns (circles size) after all regular sequences in (A) from starting pattern A, as a function of the level of punishment on previous trial. Strong punishment prevents from activating the punished branch, hence, activation either goes back to Br-0 or jumps to Br-2. (C) Probability of activation of the patterns after pattern E, that is after the regular sequences of A-B-C-D-E in (A) (probability of such sequences is indicated in subtitle). (D-F) Case of strong inhibition (λ=0.60). (D) Probability of choosing the branches from starting pattern A during trial T+1. The system takes Br-1 in 71% of the trials in the absence of any punishment (‘None’). For a weak punishment, the system activates Br-1 in 65% of trials and Br-2 in 20% of trials. Moderate punishment equalizes the probability between the two branches (41% for Br-1 and 39% for Br-2). For strong punishment, the networks activates the Br-2 only (≈0% for Br-1 vs 64% for Br-2). (E) Probability of activation of the patterns (circles size) after all regular sequences in (D) from starting pattern A, as a function of the level of punishment on previous trial. Strong punishment prevents from activating the punished branch, hence, activation either goes back to Br-0 or jumps to Br-2. (F) Probability of activation of the patterns after pattern E, that is after the regular sequences of A-B-C-D-E in (D) (probability of such sequences is indicated in subtitle).

We then assumed that the decrease in gain due to punishment remained during the trial *T* and the trial T+1, based on the possibility of long-term modification of gain [47, 48]. The effects of punishment observed immediately after punishment *during* the punished trial *T* (Fig 2E, 2F) are maintained in the following trial T+1 (Fig 4). The general behavior of the system in response to the punishment rate during trial T+1 in terms of last visited branches and patterns activated after a regular sequence is independent of the activity-dependent global inhibition, as suggested in Fig 4A–4C for λ=0.55 and Fig 4D–4E for λ=0.60. However, under weak punishment, the probability of a regular sequence of A-B-C-D-E increases with inhibition (*p*_*E*_ = 0.054 in Fig 4C vs *p*_*E*_ = 0.256 in Fig 4F), indicating that the parameter *λ* has an impact on chain length. For both inhibition levels, increasing the punishment level from weak to moderate is enough to prevent the occurrence of the sequence A-B-C-D-E. The regular sequences along branch 1 shown in Fig 4A and 4C are of A-B-C-D type.

Overall, three main network behaviors are observed depending on the level of punishment:

- In the absence of punishment, the gain is the same in all units in the two branches. In that case, the stronger synaptic connection between the branching unit 4 and unit 5 drives the network behavior. This induces a more frequent activation (choice) of branch 1 by activating patterns D then E after the initial sequence A-B-C (Fig 4A and 4B). The model reproduces an exploitation strategy that increases the probability of reward [4, 5].
- After punishment of medium intensity, the gain is decreased in units 5 and 6 (coding for pattern E) active at the time of punishment. This decreases the probability of recalling patterns D or E in the punished branch 1. This occurs immediately during the punished trial *T* (Fig 2C vs Fig 2E) and in the following trial T+1 (Fig 4B and 4E). The lower gain in the punished branch 1 makes the neurons populations less responsive to input activity coming from the initial branch 0 (patterns A-B-C). Given the stronger synaptic efficacy between the punished branch 1 and the branching node 4, the system still activates the punished branch 1 but with lower probability (Fig 4A and 4D). The balanced probabilities of selecting the two branches correspond to an exploration strategy to search for the most rewarded or less punished goals [4, 5]. System can stop activating the punished pattern E (Fig 4C and 4F).
- After strong punishment, the gain is strongly decreased in the punished units 5 and 6. After the first regular sequence A-B-C (Fig 2D), the activation of patterns along branch 1 is stopped immediately at trial *T* (Fig 2F) and is avoided at trial T+1 (Fig 4A and 4C), although this branch 1 is the most strongly (synaptically) associated to the branching node. In that case, the network can go back or switch directly to branch 2. The model reproduces an avoidance strategy that prevents from further strong punishment.

The main assumptions of our model are listed below:

- Patterns are encoded by two units and each pattern shares an active unit with at least one other pattern (overlap condition),
- The overlap condition naturally gives the set of patterns a graph structure. Here we considered the case of a ‘Y-maze’ graph, which is the simplest structure for decision making (starting from one branch the system has a choice to continue on either one of the two remaining branches).
- The synaptic coupling matrix is derived from the simple Hebb rule but each synaptic weight can weaken in time when the presynaptic neuron is active, due to STD.
- Punishment signal is immediate, constant and affects the neural gain of the units encoding for the last pattern at the end of branch 1.
- Magnitude of the gain change is proportional to the punishment.

We discuss their limitations and their extensions further down progressively.

Our previous study [31] has shown that the length of regular sequences depends on $\tau_{r}$ and *γ* (see Remark 1 above). The histograms given in S2 Fig–S4 Fig reflect this dependence through the bar height of branch 0. In particular, a smaller branch 0 bar height indicates that the system moves forward along branch 1 or branch 2, hence producing longer regular sequences for slow synapses and strong inhibition, or for fast synapses and weak inhibition. We observe an increasing preference for branch 2 proportional to the punishment rate of branch 1, likewise for strong inhibition and slow synapses.

How does branch preference after a regular sequence depends on recovery time constant of synapses and inhibition? It is important to note that synapses with small recovery time constants recover faster. This fast recovery can favor reactivation of patterns that were activated during the preceding regular sequence. Indeed, S2 Fig suggests that the patterns along branch 2 that have been visited during the preceding regular sequence are reactivated for $\tau_{r}=200$. When recovery is slow (activity for $\tau_{r}=600$ in S3 Fig and for $\tau_{r}=900$ in S4 Fig), the system rather reactivates patterns in branch 0 because synapses activated along branch 2 have not yet recovered. Increasing the punishment rate decreases the probability of activation patterns in branch 1 regardless of $\tau_{r}$ and activity-dependent inhibition *λ*.

**Fig 5:**
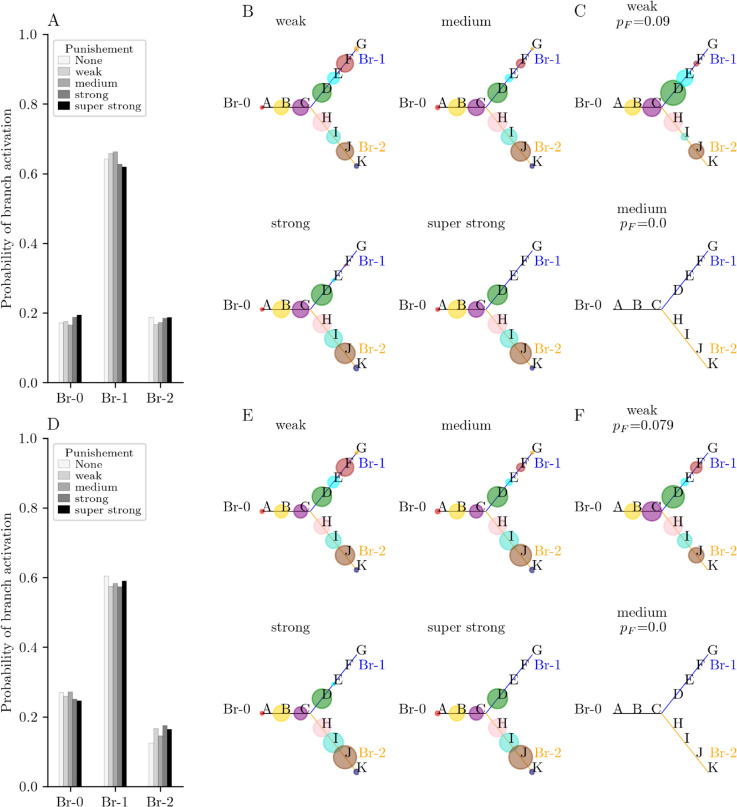
Branching behavior of a N = 12 units network at Trial T + 1. (A-C) Case of weak inhibition (λ=0.55) and (D-F) Cases of strong inhibition (λ=0.60). (A) and (D) Probability of the last activated branch during a regular sequence. For both levels of inhibition, punishment does not prevent from activating pattern D in branch 1 (Br-1) due to the stronger synaptic connection with the branching node 4 and to the fact that punishment in the preceding trial has changed gain in pattern E but D. (B) and (E) Probability of activation (circles size) of the patterns (circles size) after all regular sequences from starting pattern A in (A) and in (D), respectively, as a function of the punishment level. Increased punishment decreases the probability of activation of patterns E and F in Br-1. Strong punishment does not prevent activation of pattern D, but then the network activity either goes back to branch 0 (Br-0) or jumps to branch 2 (Br-2). (C) and (F) Probability of activation (circles size) of the patterns after pattern F, that is after the regular sequences A-B-C-D-E-F in panels (A) and (D), respectively. The probability of such a regular sequence are indicated in subtitles. Increasing punishment decreases probability of reactivation of patterns E and F.

In the model presented in Figs 2–4, punishment decreased the gain of units (5-6) directly connected to the branching unit (4) and impacted the choice behavior at unit 4 without exploring branch 1. However, sequences of activation of units preceding a feedback can be longer, leading to punishment of distant units not directly connected to the branching unit 4 (*e.g.*, 6-7). In that case the system has no information on which branch was punished when arriving at the branching unit (because unit 5 connected to unit 4 was not punished in the case of long branches). Then it should persevere in choosing the punished branch on the basis of its stronger synaptic association with the branching node. This raises the question of the mechanisms that can prevent the system from reproducing choices that lead to a punishment that is distant from the choice; distant in time as well as in intermediate units. This scenario was tested in a network with longer branches, involving *N* = 12 units and *P* = 11 learned patterns (Fig 5). Notice that this minimal configuration keeps the symmetry between branch 1 and branch 2 (*q* = 8).

When punishment arrived at patterns far from the branching node (Fig 5), the gain was decreased in units 6 and 7 and remained unchanged in all other units of the punished branch that were not active at the time of punishment (here unit 5). Then, the punished units were not directly connected to the branching node where the branch choice is made. The results show that such a distant assignment of punishment is sufficient to choose the unpunished branch in the trial T+1 (Fig 5B and 5E). The system can still activate the unpunished unit 5 at the beginning of the punished branch 1 (Fig 5A and 5D) but does not go ahead in the punished branch. For medium and higher levels of punishment, it switches branch before arriving at the punished units (6-7) (Fig 5C and 5F). We see in Fig 5C and 5F the probability of activating the pattern F during a regular sequence, hence following A-B-C-D-E-F falls to zero with increasing punishment. Finally, in the system with *N* = 12 units, the time constant determines the sequence length for λ=0.60 whereas it has less impact for λ=0.55 (S5 Fig–S7 Fig). Slower synapses decrease the probability of reactivation of pattern D on branch 1 and we observe a repartition between branch 0 and branch 2. Once again, reactivation of the punished pattern decreases considerably with the punishment rate. The activity-dependent inhibition does not impact pattern reactivation after a regular sequence. Then the network can begin in the punished branch but switches to and fully activates the other (unpunished) branch. This navigation process enables branch-switching behavior after punishment. It is not exclusive of synaptic eligibility traces [27] but does not require assignment of feedback to the whole sequence of units that were activated prior to punishment. In this way, the punishment assignment obeyed a simple mechanism that applies punishment only to units active at the time of feedback. Such a simple mechanism of assignment is made efficient even in long sequences of patterns, thanks to the network’s ability to navigate forward, backward, or jump branches in its state space, depending on the gain of the neuron population.

Interestingly, different navigation processes can be generated by different types of sequential activation in the phase space of the network. The punished state can be avoided by an activity that can either

- go back to the starting branch 0 coding for the context,
- stay at the pattern preceding the punished pattern in branch 1,
- jump directly to the unpunished branch 2.

The model exhibits elementary navigation processes within the state space of the network (*building blocks* [24]) that depend on neuronal gain. Such processes allow the network to adapt its behavioral strategy to synaptically learned reward and to the level of punishment. Taken as a whole, the results show that gain modulation switches the network behavior between exploitation and exploration behaviors:

- an approach exploitation behavior: a synaptically driven activation of the goal learned as rewarded (branch 1) rather than the goal learned as punished (branch 2) (Fig 4A, no punishment),
- an exploration behavior: a balanced selection between the goal synaptically learned as rewarded and that has been recently weakly punished (branch 1) and the goal synaptically learned as punished (branch 2) (Figs 4B and 5B, weak punishment),
- an avoidance exploitation behavior: a gain driven blocking of the strongly punished branch 1 and switch to the learned as less punished branch 2 (Figs 4B and 5B, strong punishment).

The avoidance strategy after punishment allows the system to not repeat harmful errors during trial and error learning. The level of avoidance depends on the magnitude of gain decrease, *i.e.,* of the punishment, and also on the level of inhibition *λ* in the network. This inhibitory term makes inhibition proportional to the overall level of activation of excitatory units. Interestingly, avoidance behavior increases with the level of inhibition. On the one hand, right after punishment, the system can avoid the punished branch by going backward or switching to the unpunished branch. This is almost systematic for high levels of inhibition. On the other hand, the system can persist in activating the punished branch for low levels of inhibition. In other words, the model can exhibit cautious or risky behavior depending on the level of inhibition. It would be interesting to study the mechanisms that could modulate the level of inhibition in the network. These mechanisms could be developmental or contextual, such as the rate or level of reward or punishment.

## Conclusion

From a learning point of view, the present results indicate that neuronal gain can embed knowledge about the relation between goals and outcomes. In this way, the value of neuronal gain contributes to the storage of memories of past experiences [50]. We emphasize that such memories can be updated without changes in the synaptic matrix. Such gain-based neural learning could complete synaptic learning in the alternation between exploitation, exploration and avoiding strategies. Synaptic learning allows knowledge to change rapidly and/or slowly depending on the volatility of the environment [15]. Given that gain modulation alters neuronal excitability, it could provide an alternative means of storing knowledge at the microscopic neuronal level, in addition to synapses [20, 51, 52] due to decrease of local gain by punishment signaling [42–44, 49] is capable of changing the behavior of the network at the macroscopic level without synaptic relearning.

In this study we have considered cases where punishment occurred in the branch that previously led to more reward, hence having higher synaptic efficacy with the branching node. This shows that, after punishment that strongly decreased neuronal gain, the network can completely avoid the strongly punished branch and systematically choose the unpunished one, despite its lower synaptic efficacy with the branching node. There are two possibilities after the gain decrease in the punished branch. An outcome makes possible synaptic relearning of rewards associated with the unpunished branch, until this branch has higher synaptic efficacy to the branching node than the punished one. At this point of synaptic relearning, the network would continue to choose the unpunished branch even though the gain recovers in the punished branch. Another outcome is that when the unpunished branch is not rewarded, the network would select the punished branch after gain recovery in that branch, at the risk of being punished again or a possibility that this choice leads to reward, as was the case before punishment. Therefore, gain modulation is a neuron-intrinsic learning mechanism that can work in synergy with synaptic learning to optimize the adaptation of decision making to feedback. This study shows that the ability to learn through gain modulation extends the ability to learn and adapt to changes in the environment.

We hope that these results can provide a framework for modeling and experimental approaches investigating the effects of punishment on gain modulation and goal selection without synaptic relearning. Neurophysiological experiments would be of great interest in investigating the correlations between behavior selection and gain changes in neurons coding for choices leading to punishment or reward. The current model predicts that the choices would decrease when coded by neurons with lower excitability. One could test these model predictions with genetically modified rodents subject to deficits in neuromodulators (dopamine, serotonin, noradrenalin) in a foraging tasks where feedbacks alternations require to reactivate—rather than relearn—decision strategies. Trained mice could receive reward in one branch of a Y-maze and a punishment in the other branch. Changes in feedback would require the animal to remap the branch-feedback associations one way then the other in alternating feedbacks, hence allowing to test the retention of this information across repeated alternations (through neural gain adjustment). *In vivo* measurements of neural excitability could be associated with behavioral outcome.

In this work, we assumed that the magnitude of gain change was proportional to the level of punishment. These changes can be considered constant or can also be recovered. Gain recovery, by which the gain increases progressively after punishment, can be traced indirectly by looking at decreasing levels of punishment. For example, in Figs 3–5, after a strong punishment, lower levels of punishment correspond to higher values of gain, that is, gain recovery. In this sense, the results show branch selection patterns as a function of the increasing level of punishment as well as of the increasing level of gain. Although the progressive recovery of the gain can be followed, the real-time of recovery is not represented in our model. Tracing dynamic gain recovery in terms of minutes, hours, or days would require an additional system of equations that takes the punishment signal as an argument. The main challenge here would be to control the time elapsed between the arrival of the punishment signal and the following trial while relating this time to the dynamics of neuromodulatory signaling. The latter can span in multiple spatial and temporal scales, from acute release of noradrenaline and increase in serotonin receptors to long-lasting noradrenergic hyper-reactivity, dopaminergic/serotonergic remodeling. In an extreme case, elevated amygdala activity triggered by trauma can contribute to persistance of traumatic memories and the exaggerated startle response in patients with posttraumatic stress disorder [45, 46]. Therefore, considering a constant gain modulation is a first step that mimicks biologically plausible under severe punishment like trauma, which can cause persistent changes in the network [47, 48]. Another modeling assumption is the immediate delivery of the punishment signal after pattern activation. The arrival of a late punishment signal can reduce the reactivation of the punished pattern during trial *T* as their self-excitation will decrease due to synaptic depression. So late punishment would be more in favor of exploration and avoidance strategies. There would be no difference between early and late punishment signaling during the trial T+1. Further work will investigate the effects of reward on gain modulation and its combination with punishment, as a function of the duration of the modulation of the gain before recovery.

This study focused on punishment, which is central to the rapid adaptation of decision-making processes. Cases of strong punishment are indeed special in the sense that they require rapid and efficient adaptation. Reward cases can also potentially modulate neural gain to rapidly increase choices leading to reward. In this case, the constraint of speed and absence of error is less strong, in the sense that it is less perilous to miss a reward than to reproduce a strongly punished choice. In the framework of the effects of gain on the macroscopic network behavior, intrinsic gain learning raises a number of points for further study: To what extent do the effects of gain on network behavior differ from those of synaptic learning? What conditions of intensity and frequency of reward or punishment affect transient and/or long-term behavior? What are the interactions between the variations in gain and synaptic efficacy when both change at the same time?

The model considered here was initially developed for studying priming through latching dynamics, where concepts/patterns are represented in units connected in network structures. The concepts/patterns are the neural attractors. The overlap between the patterns is essential for the spread of activity and the activation of sequences of patterns. In the present framework, patterns learned in the synaptic matrix are assumed to code for successive “places to go" in the Y-maze. The network behavior shows that it can go forward between such representations of “goals" in a branch of the maze, activate (chose) a branch when arriving at the branching node, or jump between goals in different branches. These jumps correspond to an internal switch between representations of the places to go at a “conceptual" level because an animal action could not be to jump from a branch to another. These conceptual goals are supposed to orient actual navigation actions in the maze, such as going forward in a branch or going backward to change branch. Future model refinements could couple the present network with a network whose patterns encode the sequences of actions that connect the different places to go. The encoding of “goals" in the model is at a sufficiently abstract level not limited to mazes but which can be generalized to different types of concepts in memory, such as, for example, representations of words or numbers. In this sense, the sequences produced by the network can correspond to sentences or reasoning. The model presented here has the ability to produce sequences and make decisions about which sequence to continue when it reaches a branching point as a function of previous (synaptic) learning. In case of punishment of a sequence, neuronal gain learning makes the network able to go back and jump from one sequence to another to modify its chain of reasoning. Our framework distinguishes between gradual, neuromodulator-driven reward signaling that supports one-shot learning and abrupt, gain-based punishment signaling that produces sharply defined outcomes; this contrast highlights how different neural mechanisms can shape learning speed and behavioral precision. From a general point of view, the present model shows that gain-based neuronal learning enables modulation of knowledge activated in memory for flexible decision making. This is achieved in a synapse-independent way that does not alter knowledge previously stored in the synapses. In the case of punishment, gain-based learning could then give the system the necessary time for synaptic relearning without repeating errors.

## Supporting information

S1 FigImpact of global inhibition and synaptic time constant on branching behavior at T for *N* = 10.(A) $\left(\tau_{r},\lambda )=(200,0.55\right)$, (B) $\left(\tau_{r},\lambda )=(200,0.60\right)$, (C) $\left(\tau_{r},\lambda )=(600,0.55\right)$, (D) $\left(\tau_{r},\lambda )=(600,0.60\right)$. (E) $\left(\tau_{r},\lambda )=(900,0.55\right)$, (F) $\left(\tau_{r},\lambda )=(900,0.60\right)$. The subpanels (A1-F1) summarize the branch activation probability after deactivation of pattern E that are detailed in the subpanels (A2-F2) for the unpunished case and in the subpanels (A3-F3) for different punishement levels. Circles size of the nodes in (A2-F2) and (A3-F3) is proportional to the probability of activation of the patterns. The system persists on the punished branch under weak inhibition. The global behavior of the system in response to punishment is robust to changes in $\tau_{r}$.(PDF)

S2 FigImpact of global inhibition on branching behavior at T+1 for *N* = 10 and $\tau_{r}=200$.(A-C) Activity for λ=0.55. (D-E) Activity for λ=0.60. (A) and (D) Probability of last visited branch during a regular sequence. (B) and (E) Probability of activation of the patterns after all regular sequences in (A) and in (D) as a function of the level of punishment, respectively. (C) and (E) Probability of activation of the patterns after the regular sequences of A-B-C-D-E in (A) and in (D) (probability of such sequences is indicated in subtitle), respectively. Circles size in panels (B), (C), (E) and (F) is proportional to the probability of activation of the patterns. The system reactivates the punished branch under weak inhibition and punishment strength.(PDF)

S3 FigImpact of global inhibition on branching behavior at T+1 for *N* = 10 and $\tau_{r}=600$.(A-C) Activity for λ=0.55. (D-E) Activity for λ=0.60. (A) and (D) Probability of last visited branch during a regular sequence. (B) and (E) Probability of activation of the patterns after all regular sequences in (A) and in (D) as a function of the level of punishment, respectively. (C) and (E) Probability of activation of the patterns after the regular sequences of A-B-C-D-E in (A) and in (D) (probability of such sequences is indicated in subtitle), respectively. Circles size in panels (B), (C), (E) and (F) is proportional to the probability of activation of the patterns. The system reactivates the punished branch under weak inhibition and punishment strength.(PDF)

S4 FigImpact of global inhibition on branching behavior at T+1 for *N* = 10 and $\tau_{r}=900$.(A-C) Activity for λ=0.55. (D-E) Activity for λ=0.60. (A) and (D) Probability of last visited branch during a regular sequence. (B) and (E) Probability of activation of the patterns after all regular sequences in (A) and in (D) as a function of the level of punishment, respectively. (C) and (E) Probability of activation of the patterns after the regular sequences of A-B-C-D-E in (A) and in (D) (probability of such sequences is indicated in subtitle), respectively. Circles size in panels (B), (C), (E) and (F) is proportional to the probability of activation of the patterns. The system reactivates the punished branch under weak inhibition and punishment strength.(PDF)

S5 FigImpact of global inhibition on branching behavior at T+1 for *N* = 12 and $\tau_{r}=200$.(A-C) Activity for λ=0.55. (D-E) Activity for λ=0.60. (A) and (D) Probability of last visited branch during a regular sequence. (B) and (E) Probability of activation of the patterns after all regular sequences in (A) and in (D) as a function of the level of punishment, respectively. (C) and (E) Probability of activation of the patterns after the regular sequences of A-B-C-D-E-F in (A) and in (D) (probability of such sequences is indicated in subtitle), respectively. Circles size in panels (B), (C), (E) and (F) is proportional to the probability of activation of the patterns. Punishment signal does not impact the branch activation. The system reactivates the punished pattern under weak inhibition and punishment strength.(PDF)

S6 FigImpact of global inhibition on branching behavior at T+1 for *N* = 12 and $\tau_{r}=600$.(A-C) Activity for λ=0.55. (D-E) Activity for λ=0.60. (A) and (D) Probability of last visited branch during a regular sequence. (B) and (E) Probability of activation of the patterns after all regular sequences in (A) and in (D) as a function of the level of punishment, respectively. (C) and (E) Probability of activation of the patterns after the regular sequences of A-B-C-D-E-F in (A) and in (D) (probability of such sequences is indicated in subtitle), respectively. Circles size in panels (B), (C), (E) and (F) is proportional to the probability of activation of the patterns. Punishment signal does not impact the branch activation. The system reactivates the punished pattern under weak inhibition and punishment strength.(PDF)

S7 FigImpact of global inhibition on branching behavior at T+1 for *N* = 12 and $\tau_{r}=900$.(A-C) Activity for λ=0.55. (D-E) Activity for λ=0.60. (A) and (D) Probability of last visited branch during a regular sequence. (B) and (E) Probability of activation of the patterns after all regular sequences in (A) and in (D) as a function of the level of punishment, respectively. (C) and (E) Probability of activation of the patterns after the regular sequences of A-B-C-D-E-F in (A) and in (D) (probability of such sequences is indicated in subtitle), respectively. Circles size in panels (B), (C), (E) and (F) is proportional to the probability of activation of the patterns. Punishment signal does not impact the branch activation. The system reactivates the punished pattern under weak inhibition and punishment strength.(PDF)
