# Supplementary figures and images for: Gain modulation of probabilistic selection without synaptic relearning
Source: PLoS One. 2025 Sep 30;20(9):e0333350. doi: 10.1371/journal.pone.0333350 (PMC12483250; doi:10.1371/journal.pone.0333350)

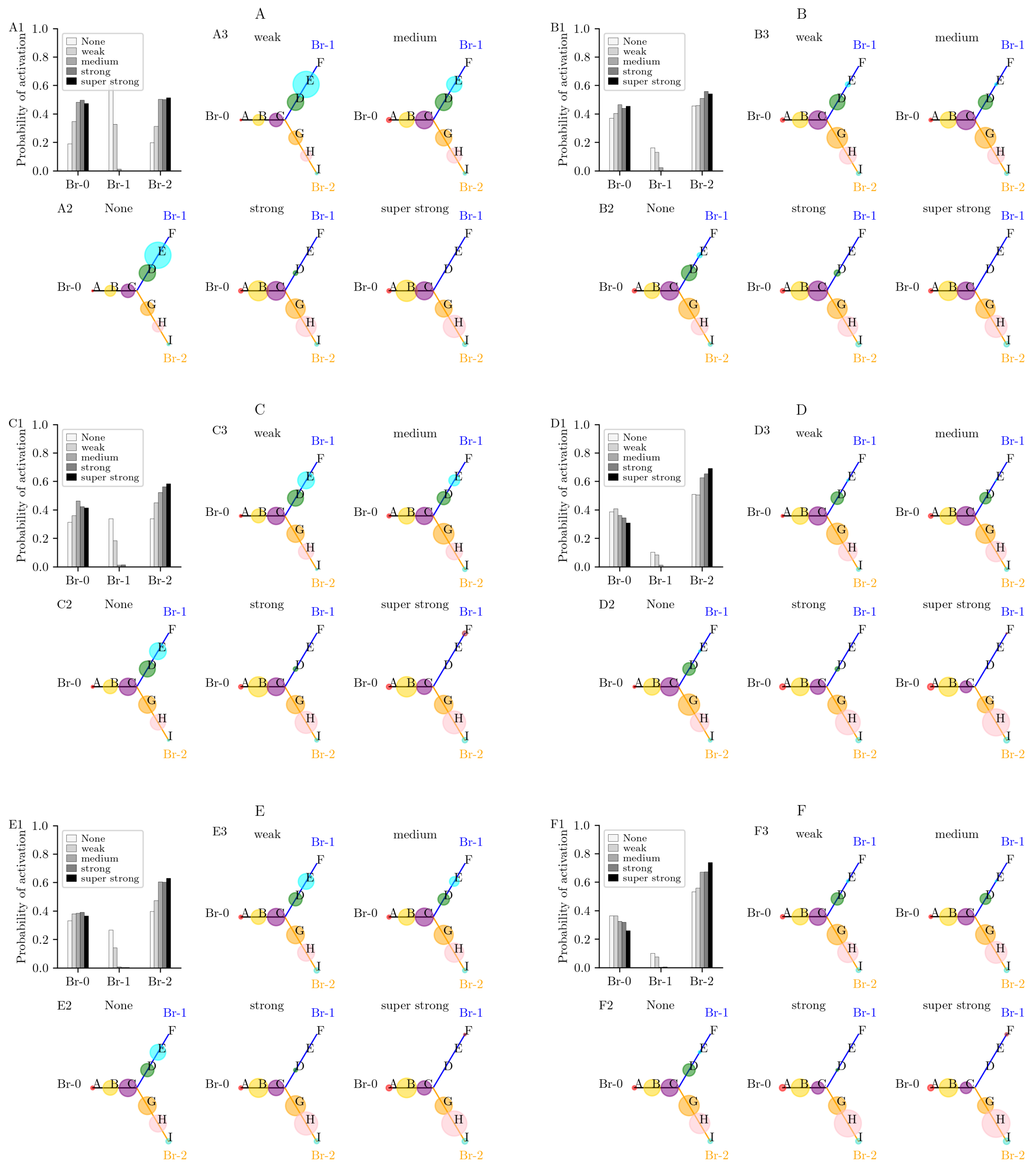

Supplement: S1 Fig — (A) (τr,λ)=(200,0.55), (B) (τr,λ)=(200,0.60), (C) (τr,λ)=(600,0.55), (D) (τr,λ)=(600,0.60). (E) (τr,λ)=(900,0.55), (F) (τr,λ)=(900,0.60). The subpanels (A1-F1) summarize the branch activation probability after deactivation of pattern E that are detailed in the subpanels (A2-F2) for the unpunished case and in the subpanels (A3-F3) for different punishement levels. Circles size of the nodes in (A2-F2) and (A3-F3) is proportional to the probability of activation of the patterns. The system persists on the punished branch under weak inhibition. The global behavior of the system in response to punishment is robust to changes in τr. (PDF) [file pone.0333350.s001.pdf]

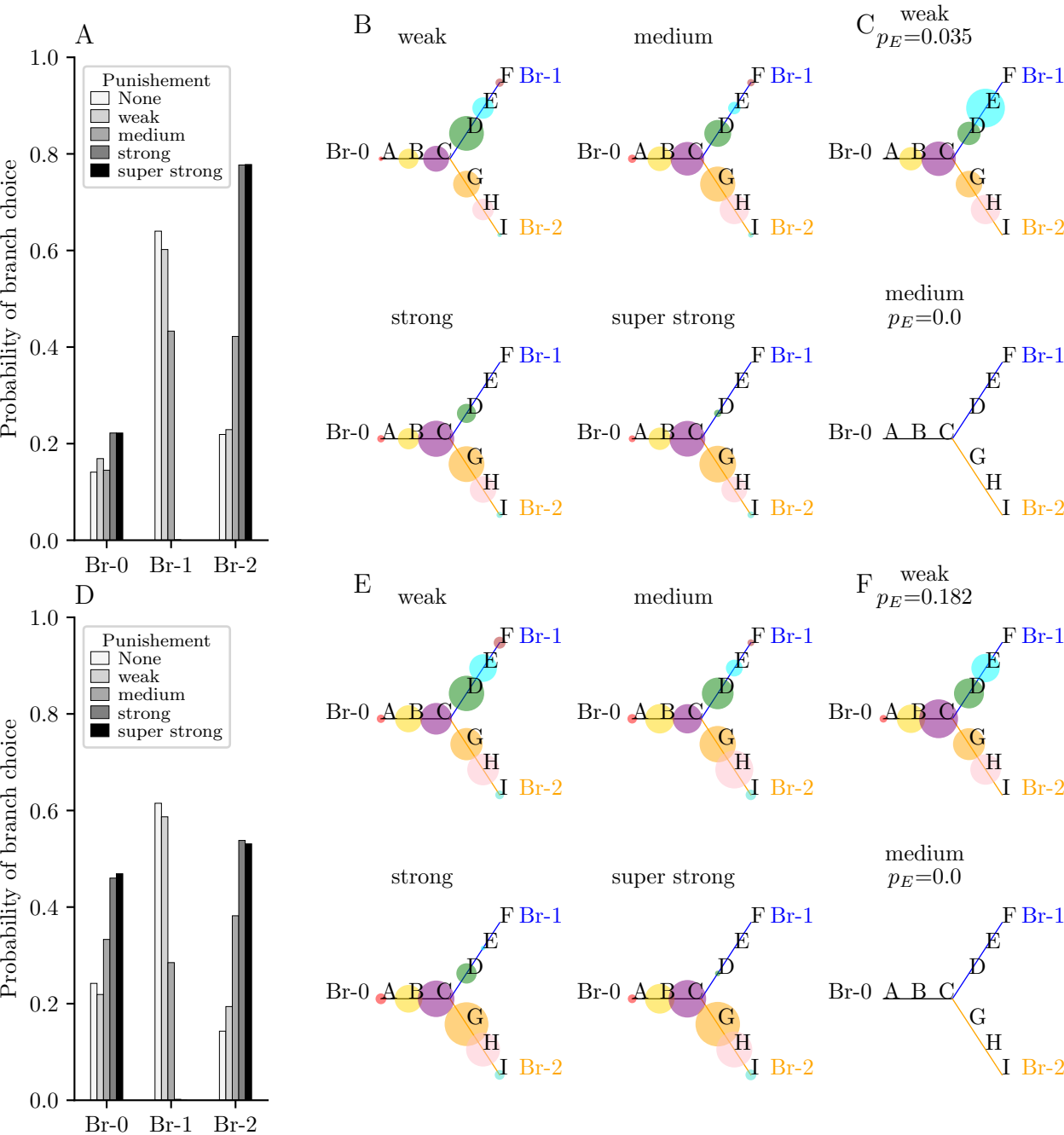

Supplement: S2 Fig — (A-C) Activity for λ=0.55. (D-E) Activity for λ=0.60. (A) and (D) Probability of last visited branch during a regular sequence. (B) and (E) Probability of activation of the patterns after all regular sequences in (A) and in (D) as a function of the level of punishment, respectively. (C) and (E) Probability of activation of the patterns after the regular sequences of A-B-C-D-E in (A) and in (D) (probability of such sequences is indicated in subtitle), respectively. Circles size in panels (B), (C), (E) and (F) is proportional to the probability of activation of the patterns. The system reactivates the punished branch under weak inhibition and punishment strength. (PDF) [file pone.0333350.s002.pdf]

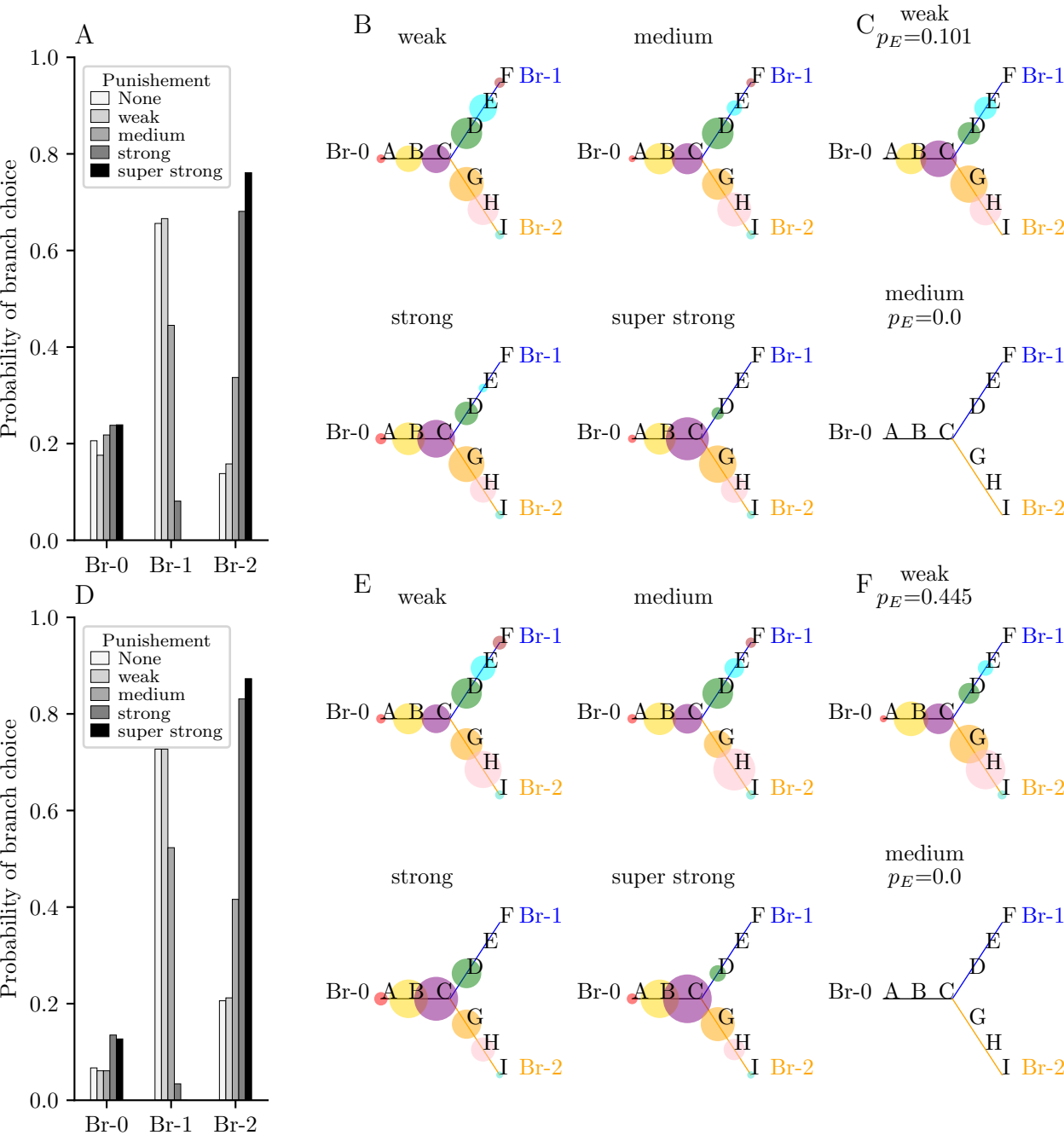

Supplement: S3 Fig — (A-C) Activity for λ=0.55. (D-E) Activity for λ=0.60. (A) and (D) Probability of last visited branch during a regular sequence. (B) and (E) Probability of activation of the patterns after all regular sequences in (A) and in (D) as a function of the level of punishment, respectively. (C) and (E) Probability of activation of the patterns after the regular sequences of A-B-C-D-E in (A) and in (D) (probability of such sequences is indicated in subtitle), respectively. Circles size in panels (B), (C), (E) and (F) is proportional to the probability of activation of the patterns. The system reactivates the punished branch under weak inhibition and punishment strength. (PDF) [file pone.0333350.s003.pdf]

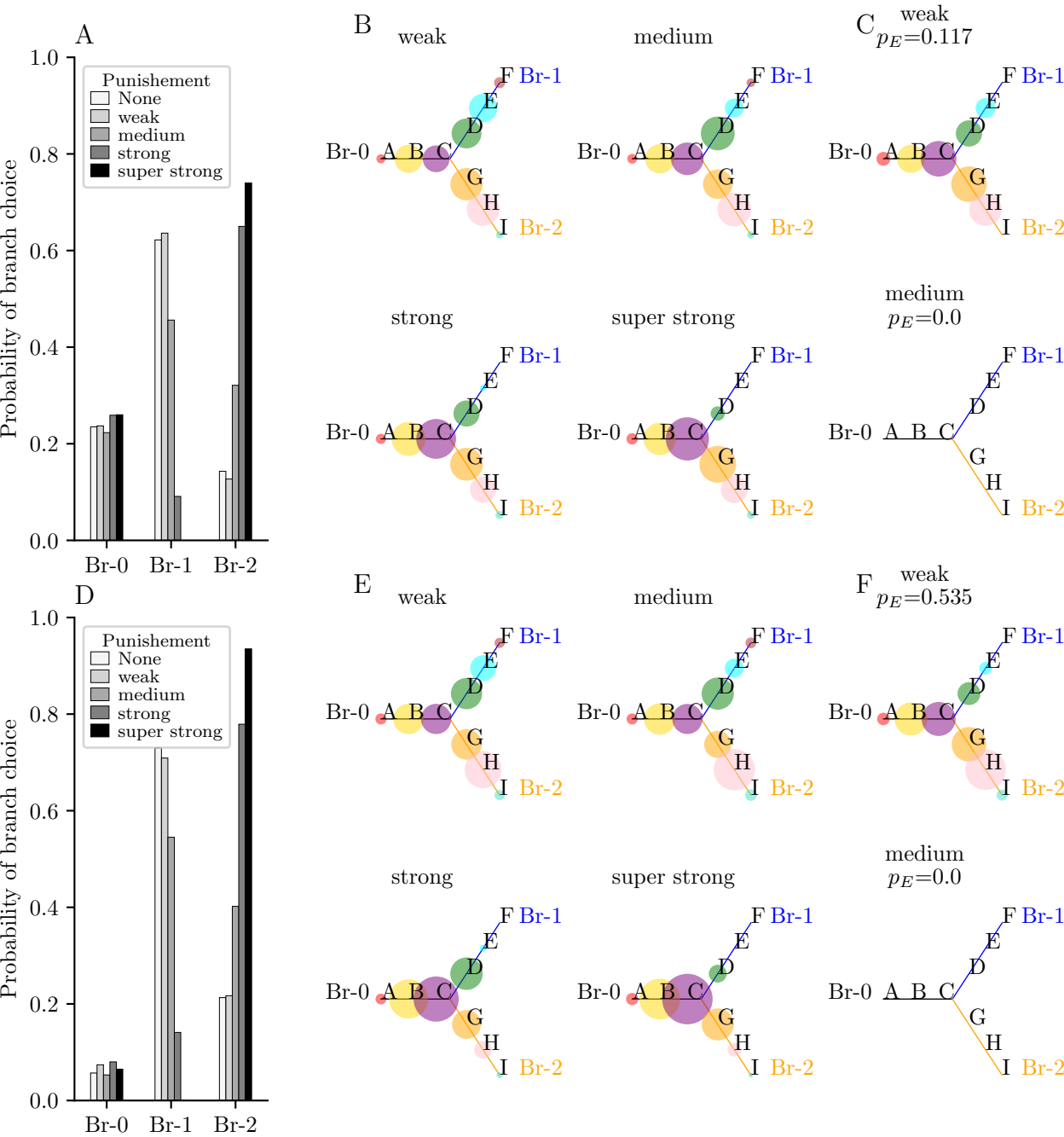

Supplement: S4 Fig — (A-C) Activity for λ=0.55. (D-E) Activity for λ=0.60. (A) and (D) Probability of last visited branch during a regular sequence. (B) and (E) Probability of activation of the patterns after all regular sequences in (A) and in (D) as a function of the level of punishment, respectively. (C) and (E) Probability of activation of the patterns after the regular sequences of A-B-C-D-E in (A) and in (D) (probability of such sequences is indicated in subtitle), respectively. Circles size in panels (B), (C), (E) and (F) is proportional to the probability of activation of the patterns. The system reactivates the punished branch under weak inhibition and punishment strength. (PDF) [file pone.0333350.s004.pdf]

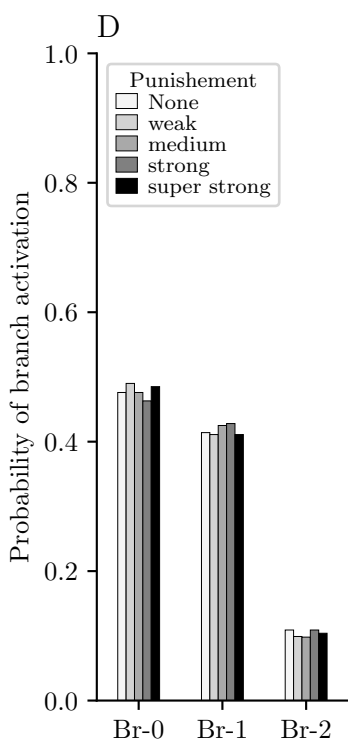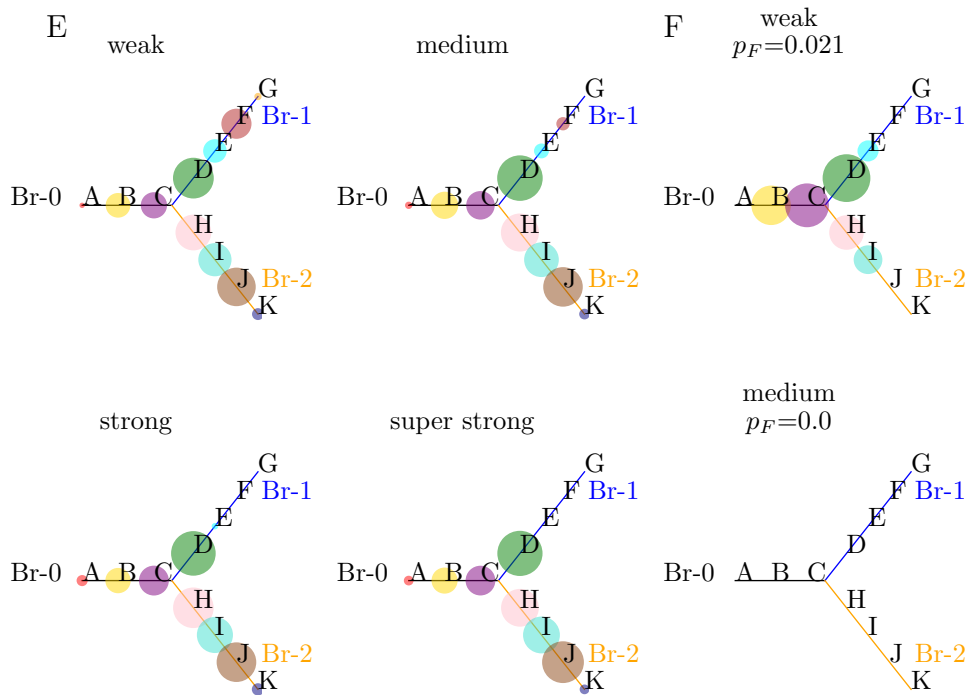

Supplement: S5 Fig — (A-C) Activity for λ=0.55. (D-E) Activity for λ=0.60. (A) and (D) Probability of last visited branch during a regular sequence. (B) and (E) Probability of activation of the patterns after all regular sequences in (A) and in (D) as a function of the level of punishment, respectively. (C) and (E) Probability of activation of the patterns after the regular sequences of A-B-C-D-E-F in (A) and in (D) (probability of such sequences is indicated in subtitle), respectively. Circles size in panels (B), (C), (E) and (F) is proportional to the probability of activation of the patterns. Punishment signal does not impact the branch activation. The system reactivates the punished pattern under weak inhibition and punishment strength. (PDF) [file pone.0333350.s005.pdf]

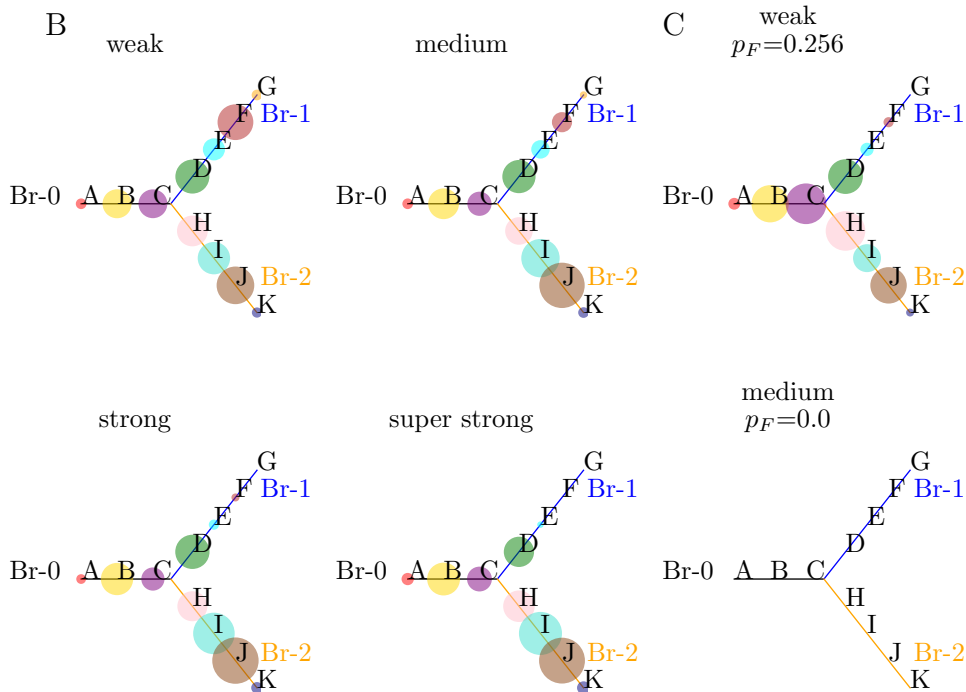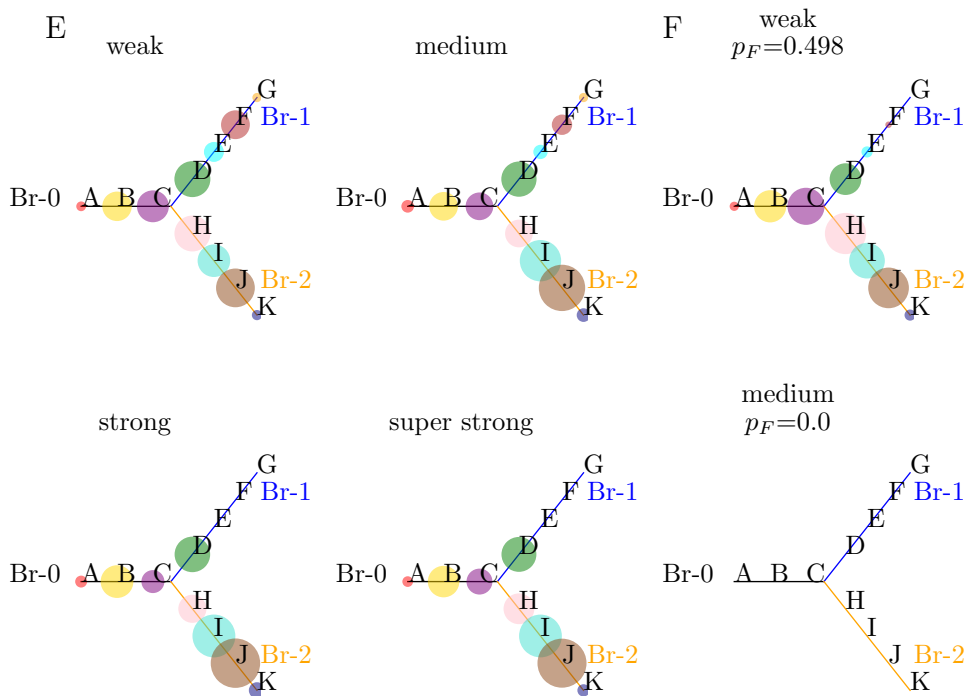

Supplement: S7 Fig — (A-C) Activity for λ=0.55. (D-E) Activity for λ=0.60. (A) and (D) Probability of last visited branch during a regular sequence. (B) and (E) Probability of activation of the patterns after all regular sequences in (A) and in (D) as a function of the level of punishment, respectively. (C) and (E) Probability of activation of the patterns after the regular sequences of A-B-C-D-E-F in (A) and in (D) (probability of such sequences is indicated in subtitle), respectively. Circles size in panels (B), (C), (E) and (F) is proportional to the probability of activation of the patterns. Punishment signal does not impact the branch activation. The system reactivates the punished pattern under weak inhibition and punishment strength. (PDF) [file pone.0333350.s007.pdf]
