# Supplementary material for: Gain modulation of probabilistic selection without synaptic relearning
Source: PLoS One. 2025 Sep 30;20(9):e0333350. doi: 10.1371/journal.pone.0333350 (PMC12483250; doi:10.1371/journal.pone.0333350)
Supplement: S6 Fig — (A-C) Activity for λ=0.55. (D-E) Activity for λ=0.60. (A) and (D) Probability of last visited branch during a regular sequence. (B) and (E) Probability of activation of the patterns after all regular sequences in (A) and in (D) as a function of the level of punishment, respectively. (C) and (E) Probability of activation of the patterns after the regular sequences of A-B-C-D-E-F in (A) and in (D) (probability of such sequences is indicated in subtitle), respectively. Circles size in panels (B), (C), (E) and (F) is proportional to the probability of activation of the patterns. Punishment signal does not impact the branch activation. The system reactivates the punished pattern under weak inhibition and punishment strength. (PDF) [file pone.0333350.s006.pdf]

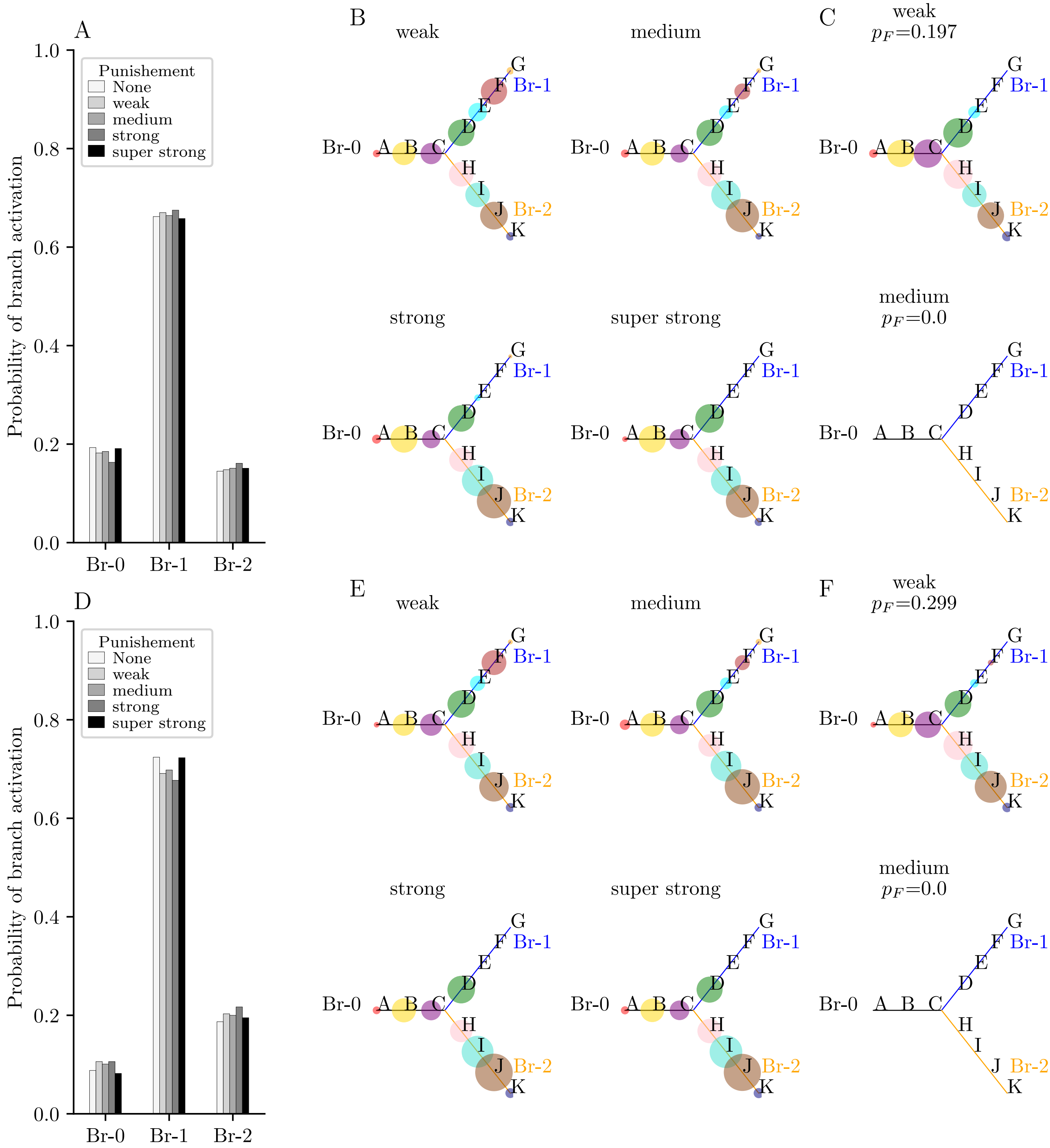

A
Probability of branch activation
1.0
0.8
0.6
0.4
0.2
0.0
Punishement
None
weak
medium
strong
super strong
Br-0
Br-1
Br-2
B
weak
medium
strong
super strong
C
weak
p_F=0.197
medium
p_F=0.0
Br-0
A
B
C
D
E
F
G
H
I
J
K
Br-1
Br-2
D
Probability of branch activation
Punishement
None
weak
medium
strong
super strong
Br-0
Br-1
Br-2
E
weak
medium
strong
super strong
F
weak
p_F=0.299
medium
p_F=0.0
